# Atraumatic splenic rupture and infection-related glomerulonephritis in a patient with infected aortic aneurysm: A case report

**DOI:** 10.1016/j.ijscr.2021.106556

**Published:** 2021-11-02

**Authors:** Shunya Kiriyama, Hisashi Imai, Nobuhisa Matsuhashi, Katsutoshi Murase, Kazuhiro Yoshida, Natsuko Suzui

**Affiliations:** a Department of Surgical Oncology, Gifu University Graduate School of Medicine, 1-1 Yanagido, Gifu 501-1194, Japan; b Department of General and Cardiothoracic Surgery, Gifu University Graduate School of Medicine, 1-1 Yanagido, Gifu 501-1194, Japan; c Department of Pathology, Gifu University Hospital, 1-1 Yanagido, Gifu 501-1194, Japan

**Keywords:** ANCA, antineutrophil cytoplasmic antibody, TEVAR, thoracic endovascular aortic repair, IE, infective endocarditis, Case report, Atraumatic splenic rupture, Infection-related splenic rupture, Infected aortic aneurysm, Infection-related glomerulonephritis

## Abstract

**Introduction:**

Atraumatic splenic rupture is very rare and the case is often difficult to determine. We report a case of atraumatic splenic rupture in a patient with an infected aortic aneurysm.

**Case presentation:**

A 40-year-old man under evaluation and treatment for renal dysfunction presented with the sudden onset of epigastric pain. The patient had a previous history of aortic arch replacement for Stanford type B aortic dissection. Contrast-enhanced computed tomography revealed intraabdominal hemorrhaging around the spleen and intrasplenic extravasation of contrast medium, and atraumatic splenic rupture was diagnosed. The patient slipped into hemorrhagic shock, and emergency splenectomy was scheduled. The histopathological diagnosis was splenic rupture with splenic infarction. The patient became febrile on postoperative day 10. Repeat contrast-enhanced computed tomography revealed enlargement of a cystic aortic aneurysm that was present prior to splenectomy. Infected aortic aneurysm was suspected, which was confirmed following thoracic endovascular aortic repair performed on postoperative day 12.

**Discussion:**

We consider that splenic rupture occurred following infected of the kidney and spleen by an infected aortic aneurysm.

**Conclusion:**

Infection should be considered as a cause in patients with atraumatic splenic rupture.

## Highlights

• Atraumatic splenic rupture was related to infection in only 27.3% of cases.
• Atraumatic splenic rupture in patients with infected aortic aneurysm is not well-documented.
• Infection-related splenic rupture should be considered in patients with acute abdominal pain.

## 1 Introduction

Splenic rupture usually occurs secondary to blunt trauma, and its management is controversial. Non-operative management is recommended in hemodynamically stable patients but carries the risk of delayed splenic rupture [1], [2]. In contrast, atraumatic splenic rupture is very rare and difficult to diagnose.

We herein report a case of atraumatic splenic rupture in a patient with an infected aortic aneurysm. This case report has been reported in line with the SCARE guidelines [3].

## 2 Case presentation

A 40-year-old man under evaluation and treatment for renal dysfunction due to suspected antineutrophil cytoplasmic antibody (ANCA)-associated vasculitis presented at our nephrology department with sudden onset of epigastric pain following three cycles of steroid pulse therapy. The results of a recent renal biopsy were unavailable. Despite constant and severe abdominal pain, the patient's abdomen was soft and flat. Serum creatinine was 5.65 mg/dl, and blood urea nitrogen was 47.9 mg/dl. Although initial vital signs were within normal ranges (heart rate, 97 beats per minute, blood pressure, 109/69 mmHg, and respiratory rate, 24 per minute), the patient's blood pressure gradually dropped and the patient became unconscious.

**Fig. 1:**
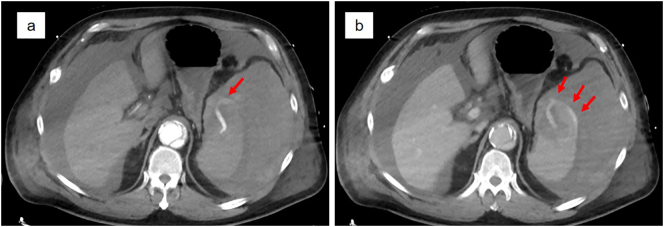
Contrast-enhanced computed tomography demonstrates intraabdominal hemorrhaging, hematomas around the spleen, and extravasation of contrast medium within the spleen (arrows) (**a**: arterial phase, **b**: equilibrium phase).

The laboratory findings were as follows: serum hemoglobin, 3.4 g/dl; hematocrit, 10.3%; leukocytes, 20,230/mm^3^; and serum C-reactive protein, 0.55 mg/dl. The patient slipped into hemorrhagic shock and was referred to the surgery department for further investigations. Contrast-enhanced computed tomography revealed intraabdominal hemorrhaging, hematomas around the spleen, and extravasation of contrast medium within the spleen (Fig. 1). Due to the lack of any recent trauma, we diagnosed atraumatic splenic rupture. The hemorrhagic shock led to cardiopulmonary arrest, from which the patient recovered spontaneously. Trans-arterial embolization was contraindicated because of a previous history of aortic arch replacement for Stanford type B aortic dissection. An anastomotic aortic aneurysm was formed following the surgery, and the celiac artery originated from the false lumen, so emergency splenectomy was scheduled. A large volume of intraabdominal blood and hematoma was removed during emergent laparotomy. Bleeding was observed from the superior border of the spleen, and intraoperative blood loss was 880 ml, including the evacuated hematoma. Because CT had confirmed arterial bleeding within the spleen, splenectomy was performed after ligation of the splenic artery.

**Fig. 2:**
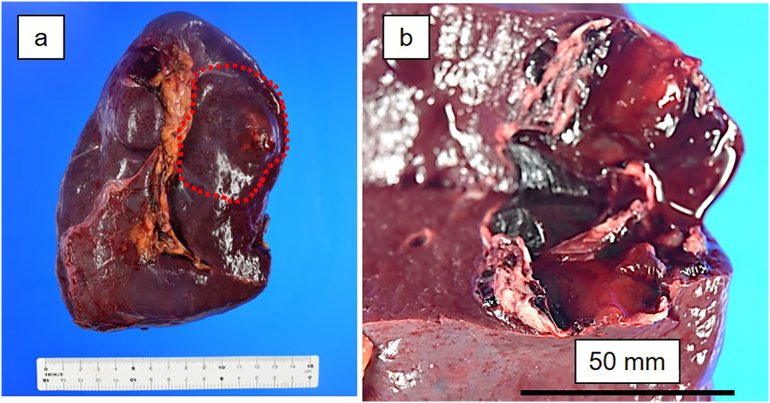
Resected specimen of the spleen. **a** An intra-splenic hematoma (red area) is seen within the specimen. **b** The hematoma is 50 mm in diameter and encapsulated by fibrous tissue. (For interpretation of the references to colour in this figure legend, the reader is referred to the web version of this article.)

**Fig. 3:**
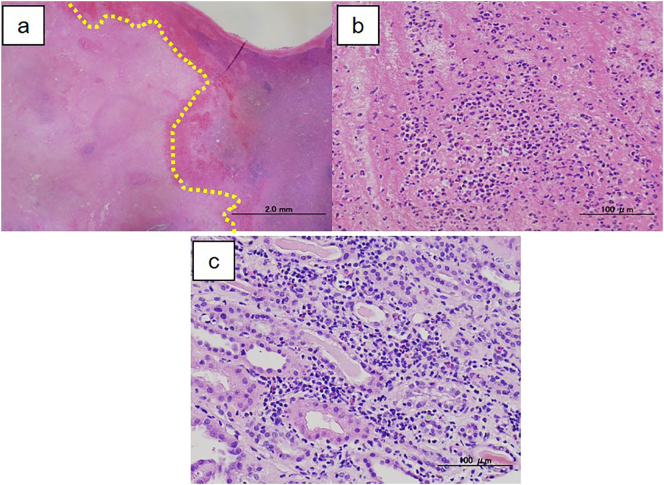
Histopathological findings (**a, b**: spleen, **c**: kidney). **a** Histopathological examination of the spleen reveals a mixture of hematomas and infarcted lesions. The non-infarcted area contains hematoma (right side of the line) and there is an infarcted area on the left. **b** The infarcted and non-infarcted lesions both show severe neutrophil infiltration. **c** The histopathological findings of the renal biopsy were of neutrophil infiltration to the intestinal cells and renal tubules.

The spleen weighed 582 g, and the resected specimen contained a splenic hematoma (Fig. 2). Pathological examination revealed a mixture of hematomas and infarcted lesions, along with severe neutrophil infiltration (Fig. 3a, b). Necrotic vessels were present in the infarcted area, with surrounding micro-abscess formation. The histopathological diagnosis was splenic rupture with splenic infarction. The histopathological findings of the earlier renal biopsy became available after the splenectomy, and revealed infiltration to the intestinal cells and renal tubules (Fig. 3c). Infiltration of plentiful inflammatory cells indicated infection-related glomerulonephritis rather than ANCA-associated vasculitis.

**Fig. 4:**
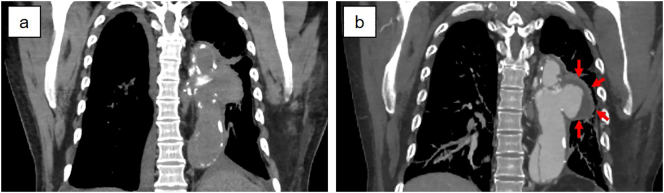
Computed tomography findings of the aortic aneurysm. **a** A cystic aortic aneurysm associated with a previous aortic arch replacement was present prior to the splenectomy. **b** Repeat computed tomography performed postoperatively reveals enlargement of the aneurysm (arrows).

The patient was ventilated postoperatively, and received dialysis in the intensive care unit. The postoperative course was uneventful, and the patient was transferred to the nephrology department on postoperative day 8 for continuation of treatment for renal failure. On postoperative day 10, the patient became febrile and infection was suspected, although blood cultures were negative. Repeat contrast-enhanced computed tomography revealing enlargement of an aortic pseudoaneurysm that had been present prior to splenectomy (Fig. 4). Infected aortic aneurysm with poorly controlled infection was suspected. Although aortic replacement is indicated for treatment of infected aortic aneurysm, the patient's general condition was poor, so thoracic endovascular aortic repair (TEVAR) was performed as a bridging therapy on postoperative day 12. The patient was then discharged on postoperative day 51, with no complications.

## 3 Discussion

It is difficult to determine the cause of atraumatic splenic rupture. Most cases are associated with anticoagulant use [4]; however, rupture can also occur in a normal spleen with no clear cause [5]. The review conducted by Renzulli et al. classified the etiology of splenic rupture into six groups: neoplastic, infectious, inflammatory, drug- and treatment-induced, mechanical, and normal spleen. They reported that the most common cause of splenic rupture was a malignant hematological disorder, such as acute myelogenous leukemia (30.4%), and that rupture was related to infection in 27.3% of cases [6].

Among the infection-related types of splenic rupture, infectious mononucleosis is most commonly responsible. Although infectious mononucleosis is a self-limiting infection, it is associated with splenomegaly and may cause splenic rupture at 21–31 days after the onset of symptoms [7].

Infectious endocarditis (IE) also carries a risk of splenic rupture, and induces systemic thromboembolism in 22%–50% of cases [8]. Cases of splenic rupture associated with IE have also been reported [9] [10]. The mechanisms underlying splenic rupture in IE patients are reported to include rupture of a splenic abscess, rupture of intrasplenic vessels with hematoma formation, and rupture of an infected splenic artery aneurysm [9]. Splenic rupture thus occurs against a background of embolized vegetations or infected splenic artery aneurysm [10] [11]. These conditions induce splenic rupture secondary to infarction. In contrast, there are no well-documented cases of atraumatic splenic rupture in patients with infected aortic aneurysm, which is a similar situation to splenic rupture with IE.

The pathological findings in the present case revealed a bloodstream infection with neutrophil infiltration and micro-abscess, indicating that the splenic rupture was associated with infection. It has been reported that due to the inflammatory response, splenic volume correlates strongly with the presence of an abdominal aortic aneurysm [12]. It is considered that the presence of an aneurysm itself causes inflammation, thus affecting the spleen and resulting in infarction. In the present patent, we initially considered a diagnosis of ANCA-associated vasculitis, but the renal biopsy findings indicated infection-related glomerulonephritis. It is likely that bacteremia caused by the infected aneurysm had infected the kidney and spleen, both of which have rich blood flow. In addition, the steroid pulse therapy started for ANCA-related vasculitis would have exacerbated the infection.

In the present rare case, we should have suspected septic embolization as soon as the pathological examination revealed micro-abscess formation, despite the lack of signs or symptoms related to infection before the patient experienced abdominal pain. We speculate that bacteremia due to the infected aortic aneurysm had formed vegetations that subsequently embolized. These minor clots then induced splenic rupture secondary to splenic infarction. Blood culture was negative in the present case; however, we cannot rule out bacteremia. As patients with infectious aneurysms do not always have positive blood cultures, repeated tests are generally performed [13].

Splenic rupture is commonly treated by surgical resection. It is our firm belief that the management of atraumatic splenic rupture is basically the same as that for traumatic cases. If the vital signs are stable, preserved management may be an option [2]. In hemodynamically stable cases, interventional radiology can also be considered for control of bleeding [14]. The present patient had an anastomotic pseudoaneurysm, and the celiac artery arose from a false lumen. We decided to perform splenectomy because of the presence of chronic abdominal malperfusion and because the patient did not respond to initial resuscitation efforts.

Endovascular treatment is a feasible option in patients with infected aortic aneurysm, but conventional surgical resection of the aneurysm is the gold-standard treatment and provides excellent outcomes [15], [16]. TEVAR was considered for our case for several reasons. First, there appeared to be adhesions around the operation site of the previous thoracotomy. Second, the patient was receiving dialysis for renal dysfunction and their general condition was considered poor following resuscitation for cardiopulmonary arrest. Finally, it would have been difficult to use an omental flap because of the history of abdominal surgery. Following treatment at our hospital, the patient had an uneventful postoperative course. The patient has received follow-up at the nephrology department since discharge and continues to undergo dialysis for renal dysfunction.

The present case indicate that the diagnosis of atraumatic splenic rupture should be kept in mind in patients with sudden onset of abdominal pain. Although our patient recovered spontaneously from cardiopulmonary arrest, a delay in the diagnosis can lead to an increase in the mortality rate. We should have evaluated the cause of rupture in the early stages, as it is very rare for rupture to occur in a normal spleen without any triggering factors.

## 4 Conclusion

We encountered a rare case of atraumatic splenic rupture caused by an infected aortic aneurysm. Septic embolization should be considered as a cause in patients with atraumatic splenic rupture.

## Ethical approval

This case report was exempted from ethical approval by our institution.

## Funding

This study received no funding support.

## CRediT authorship contribution statement

SK wrote and prepared the manuscript under the supervision of HI. IH and KM performed the surgery and managed the postoperative treatment. All authors participated in discussion about the patient's treatment. All authors have read and approved the final manuscript.

## Availability of data and material

Not applicable.

## Guarantor

Hisashi Imai.

## Consent

The patient gave their written informed consent for the publication of this case report and accompanying images.

## Registration of research studies

Not appliable.

## Declaration of competing interest

None of the authors have conflicts of interest to declare.
